# Non-invasive Brain Temperature Measurement in Acute Ischemic Stroke

**DOI:** 10.3389/fneur.2022.889214

**Published:** 2022-08-05

**Authors:** MacKenzie Horn, William K Diprose, Samuel Pichardo, Andrew Demchuk, Mohammed Almekhlafi

**Affiliations:** ^1^Department of Clinical Neurosciences, University of Calgary, Calgary, AB, Canada; ^2^Department of Medicine, University of Auckland, Auckland, New Zealand; ^3^Department of Radiology, University of Calgary, Calgary, AB, Canada; ^4^Department of Community Health Sciences, University of Calgary, Calgary, AB, Canada

**Keywords:** hypothermia, non-invasive temperature measurement, non-invasive temperature monitoring, acute stroke, magnetic resonance spectroscopy or MRS

## Abstract

Selective therapeutic hypothermia in the setting of mechanical thrombectomy (MT) is promising to further improve the outcomes of large vessel occlusion stroke. A significant limitation in applying hypothermia in this setting is the lack of real-time non-invasive brain temperature monitoring mechanism. Non-invasive brain temperature monitoring would provide important information regarding the brain temperature changes during cooling, and the factors that might influence any fluctuations. This review aims to provide appraisal of brain temperature changes during stroke, and the currently available non-invasive modalities of brain temperature measurement that have been developed and tested over the past 20 years. We cover modalities including magnetic resonance spectroscopy imaging (MRSI), radiometric thermometry, and microwave radiometry, and the evidence for their accuracy from human and animal studies. We also evaluate the feasibility of using these modalities in the acute stroke setting and potential ways for incorporating brain temperature monitoring in the stroke workflow.

## Introduction

Stroke continues to be a major cause of adult disability and death. Recent advances that achieve brain reperfusion such as mechanical thrombectomy (MT) in large vessel occlusion stroke have significantly reduced 90-day disability (1). This has also reflected favorably on the 30-day in-hospital mortality rates (2). Despite these positive indicators, ~50% of patients who undergo MT will fail to recover any better than moderate disability (1). Therefore, the need persists for useful therapies that can aid available treatments such as MT to improve stroke outcomes.

## Subjects Relevant to the Discussion

### Ischemic Stroke

#### Pathophysiology of Ischemic Stroke

**Figure 1:**
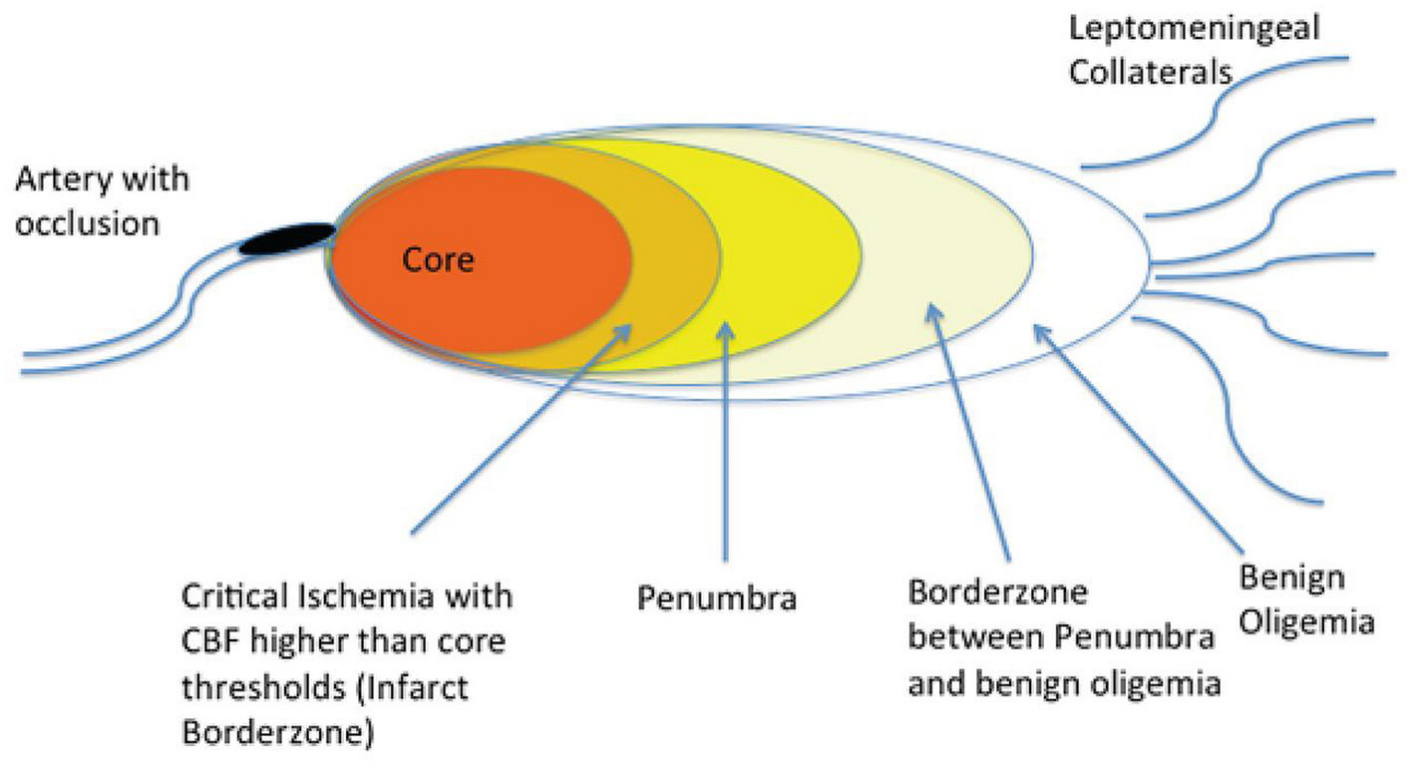
Brain regions downstream from an acute arterial occlusion. Following a cerebral arterial occlusion, the downstream brain territory evolves into an area of irreversible infarction (core), an ischemic area that is potentially salvageable if timely recanalization is achieved, and areas of somewhat reduced blood flow that can withstand ischemia even if recanalization is not achieved (benign oligemia). (Reprinted from Goyal et al. (42). Copyright (2013) by Radiology. Reprinted with permission).

Approximately 85% of all strokes are ischemic (3). An ischemic stroke is caused by an interruption in blood supply to the brain (4). Within the ischemic territory, the tissue downstream to the occlusion can be an area of benign oligemia, ischemic penumbra, or infarct core (5) (Figure 1). Since the infarct core is considered irreversibly damaged, the main target in the treatment of ischemic stroke is to preserve the ischemic penumbra as it is potentially salvageable if treated quickly. Although the oligemic region also experiences hypoperfusion, it is not at risk for damage as the penumbra is. Therefore, ischemic stroke treatment generally aims to target the penumbra (5).

#### Treatment of Ischemic Stroke

For patients who present to hospital within 4.5 h of ischemic stroke onset, intravenous thrombolysis increases the likelihood of good clinical outcomes, with faster treatment being associated with the most benefit (6). The standard of care for patients with ischemic stroke further evolved in 2015 with the publication of five trials showing significant benefit of MT and intravenous thrombolysis compared to intravenous thrombolysis alone for patients with proximal anterior circulation occlusions (1). Pooled data from these trials showed a significant reduction in disability at 90 days post-stroke for the patients who received MT in comparison to those who did not (1).

Although these results and changes in practice are promising for patients with ischemic stroke, there are still 50% of patients that will fail to recover beyond moderate disability even amongst these patients who receive MT (1). This requires continuous development of adjunct therapies to be used in the context of mechanical thrombectomy. One promising therapy is therapeutic hypothermia.

### Hypothermia

Hypothermia is a known neuroprotectant in animal models with multi-faceted and complex mechanisms of action (7). Hypothermia impacts metabolism, cerebral blood flow, glutamate release, inflammation, and apoptosis (8). This neuroprotectant has been widely researched for patients with the global brain ischemia associated with cardiac arrest. In patients with out-of-hospital cardiac arrest, hypothermia improves clinical outcomes and neurological recovery (9, 10). However, the benefit of hypothermia is less clear in the focal brain ischemia associated with stroke.

#### Brain Temperature Changes in Ischemic Stroke

Few studies have analyzed the temperature of the brain during ischemic stroke. In a study by Karaszewski et al. (11) using magnetic resonance spectroscopy imaging (MRSI) in stroke, brain temperature was higher in ischemic tissue than healthy brain tissue (11). In this study, 40 patients with acute ischemic stroke with an average National Institutes of Health Stroke Scale (NIHSS) score of 11 were included within a median time of 7 h from onset. Diffusion weighted imaging was used to obtain voxels of interest which included “definitely abnormal”, “possibly abnormal”, and their contralateral counterparts. Highest temperatures were found using MRSI in the “possibly abnormal” areas at 37.63°C and lowest in normal brain of the ipsilateral and contralateral side (37.16 and 37.22°C, respectively). This study suggests that possible penumbral tissue has the highest temperature on MRSI compared to other parts of the ischemic territory or the normal brain (11). One of the major limitations of this study is the limited capacity of diffusion weighted imaging to identify penumbral tissue. Depending on the voxel size and the size of the ischemic region, voxels could also be representative of more than one type of tissue, making it difficult to draw hard conclusions (11).

In a similar study of 40 patients with acute ischemic stroke using MRSI on admission, the average brain temperature in the core of the ischemic lesion was 38.6°C which was significantly higher than the average temperature for the contralateral hemisphere (37.9°C, *p* = 0.03) (12). There was no significant temperature difference in these brain regions when re-measured with MRSI at 5 days; the temperature in both brain hemispheres was elevated. The study also showed that higher temperatures of the contralateral normal hemisphere in the acute phase are associated with poorer outcomes. However, the elevated temperature in the ischemic region was not associated with clinical outcomes (12).

The increase in ischemic tissue temperature has implications for using therapeutic hypothermia in acute ischemic stroke. Hypothermia induces oxygen reallocation in the brain during ischemic injury (13). In addition, hypothermia decreases energy production to match the level of blood flow, therefore preserving oxygen that is already scarce during stroke, and then redistributing it. This redistribution of available oxygen can help to slow or prevent infarction (13). For patients with stroke, an increase in brain temperature disrupts many different processes within the brain. Other potential mechanisms of the benefit of hypothermia in the ischemic cascade include decreasing inflammation and excitotoxic damage (14).

#### Therapeutic Hypothermia in Stroke

##### Systemic Cooling

Hypothermia has been extensively studied in stroke animal models (15). However, whole-body (i.e., systemic) cooling, to lower the core body temperature, resulted in considerable complications in stroke patients (16). This is partly related to the side effects of systemic cooling, including pneumonia and shivering. In addition, there were considerable delays in both starting cooling and achieving target temperatures which may have counteracted any benefit from hypothermia (17). One of the ongoing studies using systemic cooling in stroke is administering endovascular cooling to achieve hypothermia fast before reperfusion while mitigating the potential for side effects (18).

##### Selective Cooling

Selective brain cooling, e.g., through intra-arterial cold infusions, aims to limit the complications of whole-body cooling while efficiently lowering the brain temperature. This method is feasible for patients planned to have MT since these patients are already undergoing an endovascular procedure with a catheter placed downstream from the affected hemisphere. Selective cooling is only able to target the affected hemisphere, and not the specific area of damage within the hemisphere. The speed at which cold fluid can reach the brain through the infusions, even if the catheter is placed downstream, prevents the entire body from cooling (19). The guide catheter used in the endovascular treatment would also facilitate the delivery of therapeutic hypothermia (19). This method has shown promising results in preliminary animal studies using rats (20, 21), swine (22), and baboon stroke models (23).

A small number of studies of stroke and non-stroke patients have demonstrated the safety of intra-arterial chilled saline infusion in humans with signals of efficacy in small studies for patients with ischemic stroke (24, 25). In a pilot study by Chen et al. (24), 26 stroke patients underwent infusion of cold isotonic saline aiming to decrease the ischemic tissue by a minimum of 2°C with little change in whole-body temperature. This pilot study demonstrated the feasibility and safety of using intra-arterial infusions during endovascular therapy (24). However, the authors did not specify how they measured the brain temperature. In a study by Wu et al. (25), 45 stroke patients who underwent endovascular therapy received intra-arterial selective cooling using 0.9% saline at 4°C and 68 controls received endovascular therapy alone. The selective cooling group had significantly smaller mean final infarct volumes after adjustment for baseline characteristics. The between-group difference was 19.1 mL (*p* = 0.038) (25). However, there was no statistically significant difference in functional outcome or clinical deterioration between groups. In both studies the chilled saline infusion occurred before and after recanalization (24, 25). Again, there was no description of brain temperature changes in this study. Diprose et al. (26), used whole brain (volumetric) echo-planar spectroscopic imaging to demonstrate that active conductive head cooling (WElkins Temperature Regulation System, 2nd Gen) reduced brain temperature by an average of 0.9°C after 80 min of cooling, whereas rectal temperature reduced by only 0.3°C, suggesting that modest non-invasive selective brain cooling is feasible (26).

#### Target Brain Temperature in Current Hypothermia Studies

To date, there have been multiple major systemic therapeutic hypothermia trials in acute ischemic stroke, with different target hypothermia temperatures and duration of cooling (Table 1). In the Cooling for Acute Ischemic Brain Damage (COOL AID) trial, patients in the cooling arm underwent cooling for 12–72 h with a goal of core body temperature maintained at 32+/– 1°C (16). In the Intravascular Cooling in the Treatment of Stroke (ICTuS) trial, patients were cooled to a temperature of 33°C for either a 12 or 24-h period (27). Similarly, in the Intravenous Thrombolysis Plus Hypothermia for Acute Treatment of Ischemic Stroke (ICTuS-L) trial, patients were cooled down to 33°C for 24-h (28). The ICTuS-2 trial, although stopped early due to changes in standard of stroke care, achieved a mean core body temperature of 35.5 +/– 1.2°C at the end of a 24-h cooling period (17).

**Table 1:** Summary of previous hypothermia clinical trials.

**Hypothermia trial**	**Sample size**	**Mean time from onset to cooling start**	**Target tissue temperature**	**Length of cooling**	**Cooling target (Core vs. Brain)**
COOL AID (16)	19 (10 hypothermia, 9 controls)	6.2 ± 1.3 h	32+/– 1°C	12–72 h	Core
ICTuS (27)	18 (All hypothermia)	8 ± 3.8 h	33°C	12 or 24 h	Core
ICTuS-L (28)	59 (28 hypothermia, 30 control, 1 not treated due to pneumonia)	Median 355 min	33°C	24 h	Core
ICTuS-2 (17)	120 (63 hypothermia, 57 control)	Median 287.6 + 65.8 min	33°C	24 h	Core

Despite the limited number of trials for therapeutic hypothermia in acute ischemic stroke, a target core-body temperature below 33°C was used for all trials and cooling occurred over a 12–72 h period (16, 17, 27, 28). Given the importance of depth and duration of hypothermia in rendering the desired neuroprotective effects of cooling, modalities that directly measure the brain temperature are equally important during hypothermia to ensure that the desired temperature is reached and maintained. With the increasing interest in selective cooling, directly measuring brain temperature is even more important as the core body temperature will be minimally impacted and thus core body temperature will not reflect brain temperature changes.

### Brain Temperature Measurement

#### Invasive Brain Temperature Measurement

It is possible to directly measure brain temperature using invasive methods (29). In a study by Huschak et al. (29), a new multiparameter catheter and a probe, for comparison, were inserted 3 cm below the cranium while the patient was under sedation (29).The multiparameter catheter system had the capability to measure brain temperature, although the device was being compared to the probe only for brain oxygen measurements (29). This method has limited applicability and feasibility in the acute stroke setting given its invasive nature.

#### The Issues With Relying on Core Body Temperature

In the setting of systemic hypothermia, core body temperature was used as an extrapolation for the brain temperature. However, literature in patients with cardiac arrest suggests that brain temperature is increased by a mean 0.34°C in comparison to average core body temperature (30). This issue also exists for patients with stroke, with literature suggesting that core body temperature is concordant with the temperature of the contralateral normal hemisphere but not the side of the stroke (31). With the current evidence suggesting that penumbral tissue increases in temperature post-stroke (31), temperature measurements that underestimates healthy vs. ischemic tissue will not be useful for patients with stroke, particularly in the context of therapeutic hypothermia. In addition, core body temperature measurement has limited applicability during selective brain hypothermia.

#### Modalities for Non-invasive Brain Temperature Measurement

Monitoring brain temperature changes in response to stroke and cooling is a limitation that could influence the successful implementation of selective intra-arterial therapeutic hypothermia. This includes temperature in both the ipsilateral and contralateral sides of the ischemic tissue and at multiple timepoints before, during, and after such treatment.

**Figure 2:**
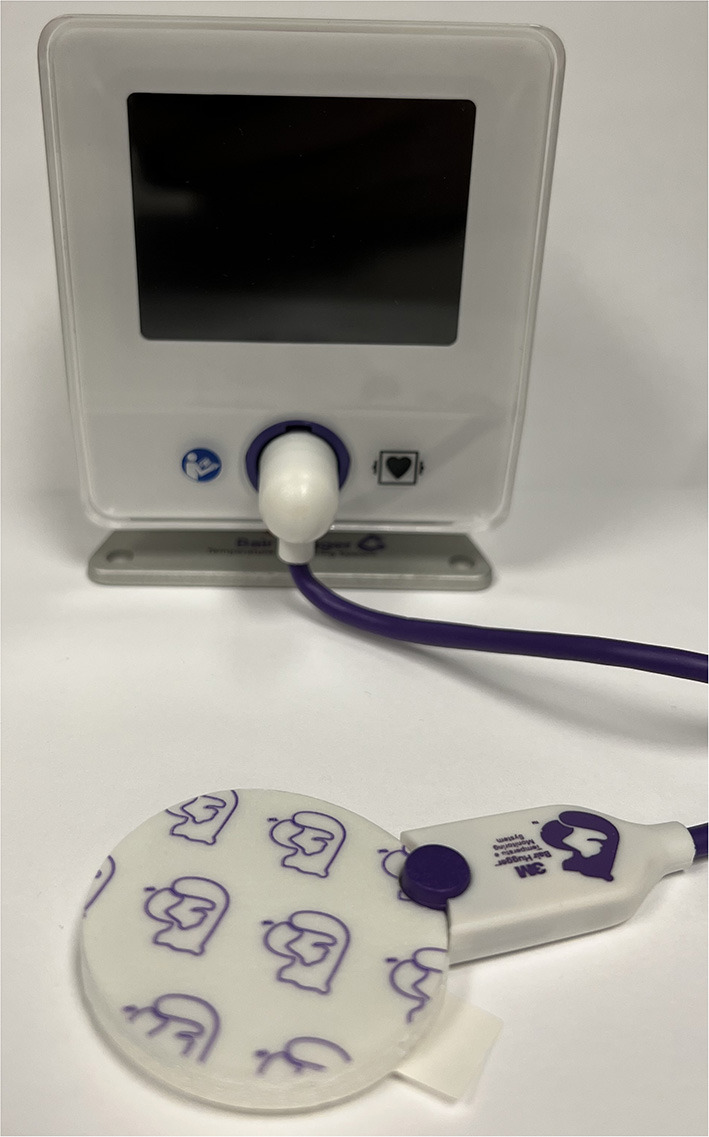
3M™ Bair Hugger™. Temperature sensors are applied to the patient's forehead.

(1) Magnetic resonance thermometry imaging
MRSI is a validated method for measuring brain temperature that has been used in few studies (11, 31–33) MRSI uses nuclear magnetic resonance to depict the chemical profile of certain metabolites within a region of interest (34).The brain has been of particular interest for MRSI clinically, because the brain is a relatively homogenous tissue (34). Studies have provided evidence for using the chemical shift of *N*-acetyl, *N*-acetyl aspartate (NAA), choline, creatine, and lactate in comparison to water to calculate brain temperature (11, 31–33). Once chemical shift differences have been found between the shift in water and the stable metabolite of choice, linear equations are used to determine temperature (35, 36). The reliability of MRSI can increase when averaging three different chemical shifts acquired from spectroscopy (36).
Early research of this technique by Corbett et al. validated the use of MRSI by comparing this technique to temperature measurements with an optical fiber probe implanted into the cerebral cortex of eight swine. The probes temperature measurements showed excellent linear agreement with MRSI (33).
Studies demonstrated validity and feasibility for using MRSI as a non-invasive approach to measure brain temperature for patients with ischemic stroke (11, 32). In a study by Marshall et al. (32), the authors validated brain temperature MRSI techniques using a phantom and then in 4 healthy volunteers. In the initial phantom test, deduced temperature from MRSI was in excellent agreement with temperature measured with a direct sensor. When using MRSI with healthy volunteers, there was no statistically significant difference in temperature between hemispheres, which was expected (32).Using similar MRSI techniques, in the same study, MRSI was conducted on 40 patients with moderate to severe stroke. The patients were imaged within 26 h from onset. Using the same validated MRSI techniques from healthy volunteers and phantom studies, they found a significant temperature difference (mean = 0.17°C elevation in abnormal tissue) between the normal brain and the lesion (*p* < 10^−3^) (32).This study used the reference metabolite as NAA in comparison to the chemical shift of water. Similarly, in another study using MRSI to determine brain temperature measurement for 40 patients with acute ischemic stroke, similar findings were evident, and NAA was also used as the reference metabolite for temperature (31).
Although MRSI is useful for brain temperature measurement, it is not feasible to use this method during endovascular cooling for acute ischemic stroke, or for continuous bedside monitoring. The availability of MRI scanners is another issue as MRI is typically not available around the clock in most centers.
(2) Radiometric thermometry
A potential alternative to MRSI for measuring brain temperature is zero-heat-flux or radiometric thermometry through sensors placed on the forehead (37, 38). Zero-heat- flux thermometry uses a temperature sensor and an electric heater to create an isothermal tunnel where the skin surface is heated to the temperature of the underlying tissue; the brain when the sensor is placed on the forehead (38, 39).The potential benefits of zero-heat-flux thermometry are significant, as it may preclude the need for more invasive monitoring.
In a study by Bass et al. (37), they used a radiometric thermometry sensor in 30 neonates with hypoxic-ischemic encephalopathy and found it effective in monitoring therapeutic hypothermia (37). The authors validated the radiometric thermometry sensors by using three mini-swine that were cooled to 33–34°C using a cooling blanket. Brain temperature was monitored using the radiometric thermometry sensors and was compared to fiber optic probes that were inserted at three depths into the swine brains. Probe measurements were similar to the temperature measurements obtained using the radiometric thermometry sensors. The study also determined the accuracy of the radiometric thermometry sensors in thirty neonates with hypoxic-ischemic encephalopathy treated with hypothermia. The rectal and esophageal temperatures were compared to radiometric thermometry sensors temperature measurements. In 60% of infants, there was an ~1°C increase in the sensor measurements as compared to rectal temperature. However, the researchers found that an increase in temperature using the sensors resulted in a significant increase in the presence of cerebral white matter injury (*p* = 0.01), which may account for the difference seen. This study suggests that radiometric thermometry sensors are safe to use but further research is needed to determine their accuracy (37).
A zero-heat-flux sensor system (3M™ Bair Hugger™) has also shown promising results for measuring brain temperature during hypothermia in pigs in comparison to thermocouple probes inserted ~1 cm below the brains surface of two pigs (38). A figure of the 3M™ Bair Hugger™ is shown in Figure 2. This study showed that the 3M™ Bair Hugger™ produced good results for pig brain temperature measurement when compared to an invasive thermocouple probe (38). When the 3M™ Bair Hugger™ and the probe were situated in the center of the forehead, there was a mean temperature difference between the two modalities of 0.3°C (38). The authors concluded that if this method can be validated in humans, zero-heat-flux thermometry presents a feasible and non-invasive tool to measure brain temperature. One limitation to these sensors is they need to be directly placed on the skin. Thus, the scalp hair will limit their placement to the forehead, however removing scalp hair can alleviate this constraint. The accuracy of zero-heat-flux thermometry in temperature measurement of the brain regions that are far away from the forehead sensor is unknown.
(3) Microwave radiometry
Microwave thermometry uses microwave frequencies to measure electromagnetic radiation occurring throughout different areas of the body (40). Its guiding principle is that the intensity of the radiation and temperature of a tissue are proportional to each other (40). In a phantom study, a radiometric antenna system was used with a brain phantom to measure blood flow and brain temperature. This proof-of-concept study was also successful at measuring temperature in a model mimicking brain tissue and showed safety and feasibility in one human volunteer. In the brain phantom, the study showed that the microwave system was able to measure conductivity and temperature up to a depth of 5 cm. The results were promising in this small study and further research is needed to assess its properties in the hypothermia setting (41).

### Non-invasive Brain Temperature Measurement and Acute Stroke Workflow

There is an ongoing need to streamline the acute stroke workflow so that one process does not delay another (Table 2). Future trials that aim to assess the effects of therapeutic hypothermia in the acute stroke setting will need to take this into consideration. To appropriately study the effects of hypothermia in the acute stroke setting, real-time monitoring of brain temperature will be necessary to ensure that the target temperature and duration of brain cooling are achieved by a given cooling method. There are limitations to MRSI in the acute setting due to availability, cost, and accessibility. Moreover, MRSI would only capture brain temperature while the patient is in the scanner which limits its use during endovascular cooling. Radiometric thermometry and microwave radiometry, if proven accurate and reliable, will have positive implications for real-time brain temperature monitoring. These sensors can be used before, during,and after endovascular cooling in parallel to the MT procedure and endovascular cooling.

**Table 2:** Brain temperature measurement and acute stroke workflow.

**Type of brain temperature measurement**	**Accuracy**	**Ease of use**	**Feasibility of use in acute stroke setting**
Temperature probes	++++	–	–
MRS	+++	+	–
Radiometric thermometry	++^*^	++	++
Microwave radiometry	Insufficient evidence to determine	++	++

^*^Data primarily from animal models only.

## Conclusions

Real-time feedback of brain temperature is necessary for testing and optimization of selective therapeutic hypothermia in patients with stroke. Brain temperature changes at key time points will also enhance the understanding of brain temperature dynamics and the safety and efficacy of reaching various temperature targets. This will also enable the comparison among various cooling methods and techniques. Available and emerging modalities for non-invasive brain temperature assessment should enable continuous monitoring throughout the acute and subacute phase with minimal interference with the acute stroke workflow. This will allow for a greater understanding of temperature fluctuations during acute ischemic stroke, and the efficiency and impact of various cooling techniques.

## Author Contributions

MH, WD, AD, and MA: contributed to the writing of the manuscript and revisions. All authors contributed to the article and approved the submitted version.

## Conflict of Interest

MA received Bair Hugger™ sensors from 3M™ as an educational grant for conduct of a clinical study. He also serves on the scientific advisory board of Palmera Medical, Inc. AD is a consultant for Medtronic, Circle NVI, and NovaSignal. The remaining authors declare that the research was conducted in the absence of any commercial or financial relationships that would be constructed as a potential conflict of interest.

## Publisher's Note

All claims expressed in this article are solely those of the authors and do not necessarily represent those of their affiliated organizations, or those of the publisher, the editors and the reviewers. Any product that may be evaluated in this article, or claim that may be made by its manufacturer, is not guaranteed or endorsed by the publisher.
